# Rabbit haemorrhagic disease: virus persistence and adaptation in Australia

**DOI:** 10.1111/eva.12195

**Published:** 2014-08-14

**Authors:** Nina I Schwensow, Brian Cooke, John Kovaliski, Ron Sinclair, David Peacock, Joerns Fickel, Simone Sommer

**Affiliations:** 1 Department of Evolutionary Genetics, Leibniz Institute for Zoo and Wildlife Research (IZW)Berlin, Germany; 2 School of Earth and Environmental Sciences, University of AdelaideAdelaide, SA, Australia; 3 Institute for Applied Ecology, University of CanberraCanberra, ACT, Australia; 4 Biosecurity SAAdelaide, SA, Australia; 5 Institute for Biochemistry and Biology, Potsdam UniversityPotsdam, Germany; 6 Institute of Experimental Ecology (M25), University of UlmUlm, Germany

**Keywords:** adaptation, calicivirus, *Oryctolagus cuniculus*, rabbit haemorrhagic disease virus epidemiology

## Abstract

In Australia, the rabbit haemorrhagic disease virus (RHDV) has been used since 1996 to reduce numbers of introduced European rabbits (*Oryctolagus cuniculus*) which have a devastating impact on the native Australian environment. RHDV causes regular, short disease outbreaks, but little is known about how the virus persists and survives between epidemics. We examined the initial spread of RHDV to show that even upon its initial spread, the virus circulated continuously on a regional scale rather than persisting at a local population level and that Australian rabbit populations are highly interconnected by virus-carrying flying vectors. Sequencing data obtained from a single rabbit population showed that the viruses that caused an epidemic each year seldom bore close genetic resemblance to those present in previous years. Together, these data suggest that RHDV survives in the Australian environment through its ability to spread amongst rabbit subpopulations. This is consistent with modelling results that indicated that in a large interconnected rabbit meta-population, RHDV should maintain high virulence, cause short, strong disease outbreaks but show low persistence in any given subpopulation. This new epidemiological framework is important for understanding virus–host co-evolution and future disease management options of pest species to secure Australia's remaining natural biodiversity.

## Introduction

Rabbit haemorrhagic disease (RHD), known since 1984 following outbreaks in domestic rabbits in China, is an acute disease affecting European rabbits *Oryctolagus cuniculus*. The causative virus (RHDV), a very small (approximately 7.4 kb) positive-sense single-stranded RNA virus belonging to the genus *Lagovirus* in the family Caliciviridae (Ohlinger et al. 1990), causes an acute and mostly fatal haemorrhagic disease specific to European rabbits. Infected susceptible rabbits die within 24–60 h *post infectionem*. RHD is regarded as a serious problem for wild rabbits in Southern Europe where rabbits represent a major food item for highly endangered higher trophic level species (e.g. European lynx, *Lynx pardinus*; Spanish Imperial Eagle, *Aquila adalberti*) as well as for commercial rabbit producers. In contrast, it has been used as a biological control agent in Australia where introduced wild European rabbits are a severe pest of agriculture and the environment (Delibes-Mateos et al. 2008; Gong et al. 2009; Abrantes et al. 2012).

Rabbit haemorrhagic disease was introduced into high security quarantine in Australia and, after several years of laboratory testing to ascertain its specificity for European rabbits, experiments commenced in quarantine compounds on Wardang Island 4 km off the coast of South Australia. The virus' escape to the mainland during testing of its field efficacy was embarrassing for the institutions involved, but it quickly became obvious that the virus could spread over long distances and was highly effective in reducing Australia's rabbit populations. Nation-wide, rabbit numbers fell by 60% and declines were even higher in arid areas where no other methods of controlling rabbits are economically feasible (Bowen and Read 1998; Mutze et al. 1998).

The escape of the virus, effectively from a single point source, provided information on its rapid continent-wide spread (Kovaliski 1998) and drew attention to the likely role of insects in its transmission (Fenner and Fantini 1999). This was supported both by modelling of movements of carrion-feeding flies at the time of the escape (Wardhaugh and Rochester 1996) and by laboratory experiments which investigated the survival and excretion of virus ingested by blowflies that fed on RHD infected rabbit liver (Asgari et al. 1999). Fly spots, that is, faeces or regurgita contained enough virus particles to infect a rabbit and if deposited on vegetation, could be readily ingested. Viable virus survived for up to 9 days in flies. Field experiments again supported the idea that flies could spread virus among rabbits (Barratt et al. 1998) after the virus was illegally introduced into New Zealand (O'Hara 2006).

Rabbit haemorrhagic disease virus has regularly caused natural disease outbreaks in the field in Australia for nearly two decades. Virus samples from dead rabbits found during recurrent RHD outbreaks have been used to calculate rates of genetic change (rate of nucleotide substitution per year) and show how virus diversity has increased over time (Kovaliski et al. 2014). Early field epidemiological studies showed that RHD epizootics occurred annually or occasionally every second year in most rabbit populations although their impact on rabbit populations varied regionally (Henzell et al. 2002). This variable impact is now partly explained by the presence of a nonpathogenic rabbit calicivirus (RCV-A1) which is most prevalent in cooler wetter parts of south-eastern and south-western Australia. RCV-A1 partially and temporarily immunizes rabbits against the full impact of RHD (Jahnke et al. 2010; Liu et al. 2012; Strive et al. 2013).

Despite this increasing epidemiological knowledge, there has been little headway made in developing a unifying concept of how RHDV survives, circulates in the environment and causes recurrent outbreaks. Linking theoretical co-evolutionary scenarios of disease evolution and environmentally driven virus adaptation using practical field observations is essential for developing a sound epidemiological and evolutionary framework.

A number of evolutionary possibilities have previously been suggested. Soon after RHDV was described and sequenced, other virus variants were rapidly discovered. The amplification of recent RHDV strains from 50-year-old sera led to the hypothesis that RHDV might have both virulent and avirulent modes of spread (Moss et al. 2002). Nonetheless, this theory was largely dismissed when calculations of the substitution rates demonstrated that these sequences were most likely contaminations with modern strains (Kerr et al. 2009), and the assumed rate of nucleotide substitution as calculated by those authors was later found to be reduced by 65% owing to the inclusion of a misdated sample in initial calculations (Hicks and Duffy 2012). The nucleotide substitution rates of 1.50–2.34 × 10^−3^ substitutions/base/year (Hicks and Duffy 2012) have subsequently again been surpassed by those obtained by direct measurement of change (3.3–4.7 × 10^−3^) following the introduction of the single RHDV variant in Australia (Kovaliski et al. 2014), which was derived from the Czech CAPM-V351 strain (GenBank Accession Number U54983) and produced for field release by the Elizabeth Macarthur Agricultural Institute (EMAI), New South Wales.

It was also reported that complete RHDV genome can be recovered from rabbits that survive RHD leading to the suggestion that virus genomic material might persist in live rabbits enabling complete virus to be shed from time to time thereby initiating new epizootics (Forrester et al. 2003). However, attempts to show that this persistent viral RNA is still infective or can be reactivated using strong immune-suppressant drugs have failed so far (Shien et al. 2000; Forrester et al. 2003; Gall and Schirrmeier 2006; Gall et al. 2007). Even if some shedding were possible, it would still need to be demonstrated that this occurred commonly and resulted in the release of sufficient quantities of viable, infective virus to be of importance in field epidemiology.

Recently, Kovaliski et al. (2014) have cast further doubt on this idea of reactivation of persistent virus. They showed that virus samples collected from a limited number of carcasses from a relatively isolated rabbit population at Turretfield, South Australia, in 1 year were not the antecedents of viruses collected in the next. In other words, the virus that spread in a given year was unlikely to have persisted in individual rabbits but instead appeared to be introduced afresh each year. This observation opens the way for proposing that the virus simply manages to survive somewhere on a wide regional scale, even if hard to detect at times, and spreads back through rabbit subpopulations when susceptible rabbits are recruited as a result of seasonal breeding and when there are suitable conditions for flies to spread virus.

With this in mind, we have re-examined data collected during the first years as RHD spread through Australia. We critically review (i) the observed rate of initial spread, (ii) the rate of spread relative to the distribution and distance between recorded rabbit populations and (iii) the seasonal distribution of disease outbreaks on a regional scale. Building upon the results of Kovaliski et al. (2014), who had investigated relatively few virus samples from Turretfield/South Australia, we more thoroughly sampled the available archived tissues from rabbit carcasses from this population. We sequenced viruses from more individual rabbits as well as samples from intermediate years that had not been sampled before. This allowed us to test the idea set out by Kovaliski et al. (2014) more rigorously to see whether a larger data set consistently supported the idea that RHDV is re-introduced each year. We also tried to ascertain whether there is evidence that more than one distinct virus variant can be detected during a given outbreak.

We considered that epidemiological processes could not be determined by simple virus–host interactions alone. Connectivity between rabbit populations (in terms of virus spread), for example, is clearly dependent on insect vectors, most probably flies, and not on rabbit movement between territories. If vector-based connectivity between rabbit populations is strong, virus variants would not necessarily compete within rabbit subpopulations, but rather on a wider regional scale. Natural selection would favour virus variants with the capacity to spread ahead of others into new rabbit subpopulations, where a previous variant had died out, and quickly infect all susceptible rabbits.

We see the results from these field observations, molecular and epidemiological studies as valuable for testing the various scenarios proposed earlier by Fouchet et al. (2009) to anticipate the likely co-evolution of virus virulence and rabbit resistance. Those authors argued that virus competition and evolution within small subpopulations of rabbits was the most likely scenario in Europe, but they also explored models that assumed infinite host populations and high connectivity between subpopulations. Under this second scenario, they reasoned that successful virus variants would be selected for high virulence and have a high capacity to spread from one rabbit population to another. These variants would cause short, strong disease outbreaks but not persist for long. To us, this second scenario most closely fits the observed field epidemiology of RHDV in South Australia, so providing a general epidemiological model that should be considered for planning future rabbit conservation in Europe, as well as biocontrol strategies of this pest species in Australia.

## Materials and methods

### Study region and data collection

**Figure 1:**
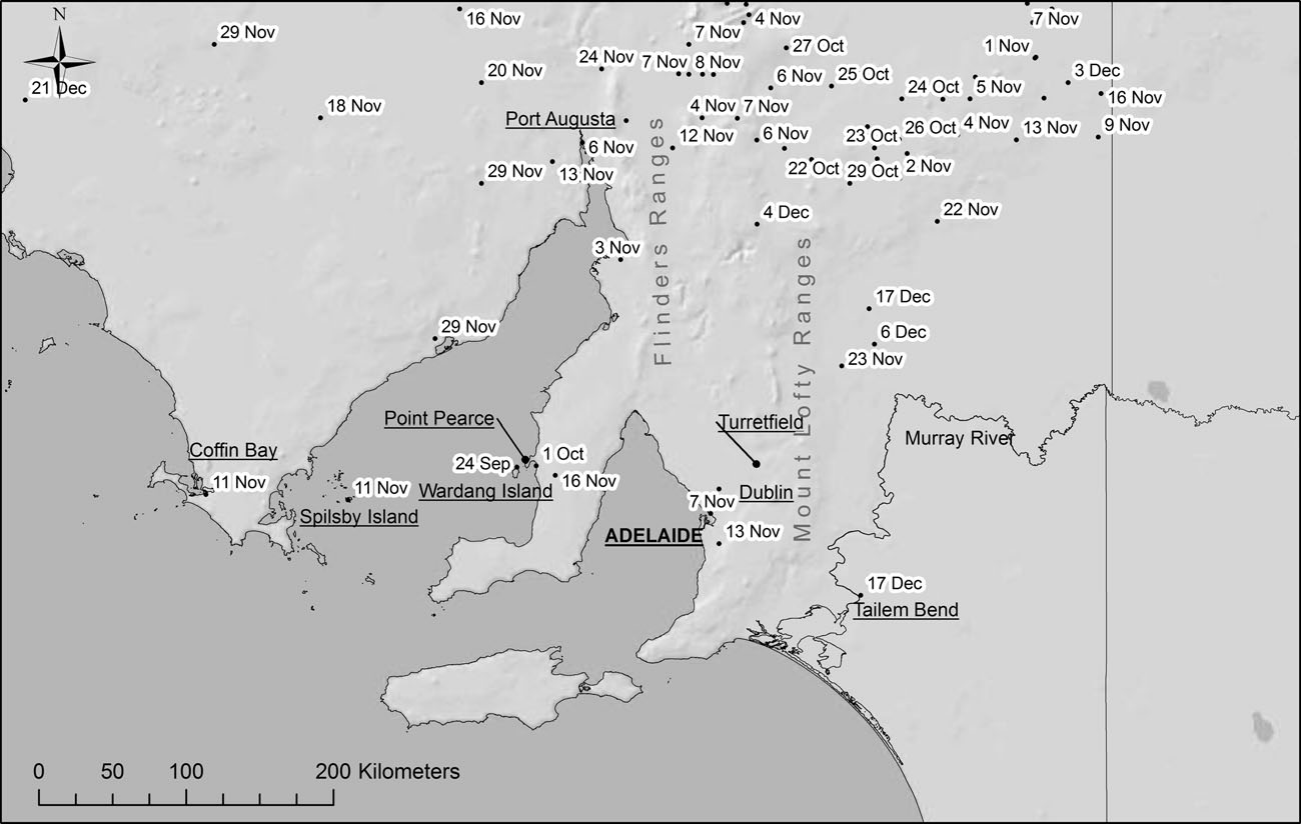
Map of the locations and estimated day of virus arrival after the escape of rabbit haemorrhagic disease virus (RHDV) from Wardang Island and during its spread through South Australia in 1995.

The initial spread of RHD from Wardang Island to other parts of Australia was monitored using reports of disease outbreaks confirmed by the detection of virus in samples of dead rabbits obtained from each outbreak site. A database was set up and is maintained by Biosecurity South Australia to manage information on dates of collection of dead rabbits, verification of the presence of RHDV through laboratory tests, recording of the exact location of outbreaks (latitude and longitude) to facilitate mapping and measurement of rates of spread (Kovaliski 1998). In this paper, we have confined our investigations to populations within a smaller region of South Australia centred on the Adelaide Hills (Mount Lofty Ranges) but also including Wardang Island (which was the source of the virus) and the Murray River region to the east, that is, the region shown in Fig.1. To put this in perspective, it should be noted that the area chosen encompasses roughly about 40% of the area of Spain, so we are considering epidemiological events at a scale somewhere between a provincial level and a country-wide scale as far as Europe is concerned.

We used dates of estimated first arrival of RHDV at specific sites for calculating rates of virus spread rather than dates on which dead rabbits were collected (as in Fenner and Fantini 1999) or the date when the presence of virus was confirmed by laboratory tests (as in Kovaliski 1998). The latter dates sometimes lagged considerably behind events. As an example, Fenner and Fantini (1999, table 11.3) showed that the first rabbit on the Australian mainland that was confirmed to have died from RHD was picked up on 12 October 1995. After allowing for the infection period, the rabbit's state of decomposition and the extent and rate of local spread of the virus revealed by subsequent investigation, it was clear that the virus had arrived earlier, on about 1 October 1995. These virus arrival dates were estimated by Biosecurity SA staff in 1995 and stored in the database used here.

To construct a map of all presently known rabbit populations in the region, we combined information available in the Biosecurity SA database, with Australia-wide records collated by Dr D. Berman associated with release of Spanish rabbit fleas (*Xenopsylla cunicularis*) as vectors of myxomatosis, data from experimental sites for rabbit control studies, state fauna surveys and results from the Rabbit Scan project (http://www.feralscan.org.au/rabbitscan). The Rabbit Scan project is a website for community *ad hoc* reporting of sites where rabbits are problematic. Using those data, we were able to consider in detail (i) the spatial distances between rabbit populations in which initial RHD outbreaks occurred and the minimum rate at which the virus first spread (km day^−1^) through the region; (ii) the proximity of known rabbit populations to each other relative to rates of virus spread; and (iii) the occurrence of RHD outbreaks throughout the year within the region selected. It has to be noted that the map reflects only the minimum density of rabbit populations. It is thus conservative; there are many more rabbit populations than those recorded with accurate coordinates.

Virus samples for sequencing were collected from our epidemiological study site at the Turretfield Research Centre (34°33′S, 138°50′E), which is located 50 km north of Adelaide, South Australia. The rabbit population is 3–4 km distant from other rabbit populations, with only a few scattered rabbit warrens in between (Peacock and Sinclair 2009). In late October 1996, rabbits live-trapped on the site were inoculated with the official biocontrol RHDV strain and released. Since then, a continuous capture-mark-recapture study has been conducted on a regular basis. Rabbits have been live-trapped every 8–10 weeks, but the site was visited more frequently, especially in spring when disease outbreaks were likely, to search for dead rabbits and often daily once an outbreak of RHD was detected. The numbers of rabbit carcasses collected daily and verified to have died from RHD provided a picture of the duration and intensity of outbreaks. This work was carried out with full animal experimentation ethics approval (PIRSA AEC 09/03) although in this particular study, the work only involved sampling rabbits that had naturally died from the disease.

Liver, spleen or bone marrow samples from dead rabbits were collected and frozen for later analysis. Between 1996 and 2006, samples were not available from every year. At times, few rabbit carcasses were collected and RHD outbreaks did not occur every year (Mutze et al. 2014). However, a large number of samples were available from 1999, and used to indicate changes in the virus in the first 3 years after virus was known to have been released on the site. Since 2006, RHD outbreaks have occurred annually and more samples with high-quality viral RNA have become available for sequencing. In total, RNA was extracted from 63 individual rabbits and sequenced to provide a picture of long-term changes in RHDV within a single rabbit population.

### Laboratory methods

Total RNA was extracted using approximately 30 mg of liver, marrow or spleen (whichever was available). Tissue was placed in a 2-mL tube containing 500 μL QIAzol lysis reagent (Qiagen, Hilden, Germany). The tissue was disrupted in a Precellys homogenizer (PeqLab, Erlangen, Germany) using Ø1.4 mm ceramic beads at 5000 rpm for 2 × 10 s. RNA isolation followed the QIAzol lysis buffer protocol (Qiagen). Total RNA was dissolved in 87.5 μL RNAse-free water, cleaned-up using RNeasy spin columns (Qiagen) and finally eluted in 60 μL of RNAse-free water. We measured concentration and purity of the RNA using a Nanodrop 1000 (Thermo Scientific, Waltham, MA, USA). For each sample, we constructed two independent first-strand cDNA libraries using up to 5 μg of total RNA, Oligo-dT_18_ primers and the Revert AidTM H Minus First Strand cDNA Synthesis Kit (Fermentas/Thermo Scientific, Waltham, MA, USA) as instructed in the manufacturer's protocol.

Virus presence was confirmed by polymerase chain reaction (PCR). We amplified a 504-bp fragment (nucleotide position 872–1376 of the RHD virus capsid protein gene) using forward primer rhdNS1f (5′-CGTTTGCCGACATTGACCA-3′) starting at capsid position 872 and reverse primer rhdNS1r (5′-GTGTTCTTACCCACAGGTGC-3′) which ends at capsid position 1357. This fragment covers most of the P2 region (aa 287–449) which forms an external loop of the virus capsid and contains the less conservative regions V1–V6 that are thought to be important for antibody interaction (Wang et al. 2013). We did not fully sequence virus capsid because we considered it would be more valuable to have sequences from as many virus variants as possible but, to meet project constraints, restrict sequences to what is currently considered one of the most antigenetically important and variable parts of the capsid genome. This region has an elevated d*N*/d*S* ratio when compared to the whole capsid (data not shown), and thus, it was logical to concentrate on this most variable section of virus genome.

Amplifications were run in a final volume of 25 μL including 10–100 ng cDNA, 15 pmol of each primer, 0.8 mm dNTP mix, 1× buffer and 0.5 U Taq polymerase (MP Biomedicals, Irvine, CA, USA). The thermal profile consisted of an initial denaturation at 94°C for 1 min, 35 cycles of 15 s at 94°C, 30 s at 55°C, 1 min at 72°C with a final extension period at 72°C for 10 min in a T Gradient (Biometra, Goettingen, Germany) or T Professional Thermocycler (Biometra). Positive samples were sequenced bidirectionally using BigDye®; Thermo Fisher Scientific Terminator v.3.1 Cycle Sequencing Kit (Applied Biosystems®; Thermo Fisher Scientific) and analysed on ABI PRISM 3110xl Automated Genetic Analyser (Applied Biosystems®).

### Data analyses

Database information was extracted on the number of RHDV outbreaks recorded each month from the time RHDV escaped off Wardang Island in October 1995 until December 1996 to determine whether there was credible evidence that the virus could potentially survive by circulating continuously through a wide regional landscape. Database information on dates and locations of RHD outbreaks during these early phases of spread was examined using ESRI®Arc Map 10 (Redlands, CA, USA) to determine how quickly RHDV had spread from Wardang Island to more distant rabbit populations (km week^−1^). The values obtained were then considered together with maps of the distribution of all known rabbit populations throughout the study region to ask whether or not RHDV could readily move from one rabbit subpopulation to another.

To examine patterns of sequence variation, nucleotide sequences were edited based on their forward and reverse chromatograms and *in silico* translated into deduced amino acid sequences. We used the maximum likelihood method to reconstruct the phylogenetic relationships among all virus sequences (henceforth called RHDV variants) isolated from rabbits found dead at Turretfield. We used the general time reversible model allowing for a proportion of invariant sites and a gamma distribution of among-site variation with five categories. Bootstrapping based on 1000 resampled replicate ML trees (utilizing NNI branch-swapping) was used to estimate the support for individual nodes. Alignment and molecular evolutionary and phylogenetic analyses were conducted using MEGA version 6 (Tamura et al. 2013) and the tools implemented therein.

## Results

### Analysis of maps and other data on the initial virus spread

The escape of RHDV from quarantine compounds on Wardang Island, where it was being assessed for efficacy in controlling rabbits, began with its spread between rabbit pens on 23 September 1995. The first dead rabbit outside the quarantine area on Wardang Island had presumably become infected on 24 September 1995, and further dead rabbits were then found on the Island for about a month. The first dead rabbit confirmed to have RHD on the mainland was discovered on 12 October 1995 on Point Pearce, close to the Island (Fig.1). However, RHD was estimated to have arrived on that site on 1 October 1995 some 11 days earlier while the virus was spreading among rabbits on Wardang Island. Soon after, it reached Yunta and Gum Creek in the north-east of South Australia, two widely separated sites over 300 km from Wardang Island (Kovaliski 1998). The first dead rabbits were detected on 25 and 26 October although subsequent considerations based on the extent of spread and weather conditions suitable for long-distance spread on flies indicated it had arrived there by at least 22 October 1995 and possibly as early as 12–13 October 1995 (Fenner and Fantini 1999, pp. 256–257).

It was considered to have reached Port Augusta about 230 km north of Wardang Island on about 6 November 1995, and by 11 November 1999, it had even spread to Spilsby Island, an uninhabited island about 20 km off the coast of Eyre Peninsula and about 100 km west of Wardang Island. On the same day, an RHD outbreak was recorded at Coffin Bay on Eyre Peninsula, about 175 km west of Wardang Island. The virus also spread eastward from Wardang Island and on the 7 November 1995 reached Dublin (about 30 km north of Adelaide) suggesting that it either had spread about 60 km across the sea or that it had taken a much longer route along the coastline. By 17 December 1995, it had reached Tailem Bend on the Murray River about 210 km east of Wardang Island (Table1).

**Table 1:** The dates of first rabbit haemorrhagic disease outbreaks in South Australian rabbit populations.

Population	Date	Days since earliest escape	Distance to Wardang Island (km)	Per day (km)	Distance per week (km)
Southern Flinders Ranges	22 October 1995	28	250	9	63
Port Augusta	6 November 1995	43	230	5	37
Dublin, SA	7 November 1995	44	110	3	18
Spilsby Island	11 November 1995	48	100	2	15
Coffin Bay	11 November 1995	48	175	4	26
Tailem Bend	17 December 1995	84	210	3	18

The number of days since escape is the number of days since the 24 September 1995 when the first dead rabbit outside the quarantine pens on Wardang Island became infected.

These records indicate a rate of spread of 2–9 km day^−1^ or about 15–60 km week^−1^ although the spread across the Adelaide region is at the lower end of this scale (about 20 km week^−1^). This must be taken as a minimum rate, because, if RHDV spread from one rabbit population to another, it is unlikely to have spread in a direct line between any two localities. It also implies the virus was transferred by flying insects or birds over 20–100 km of sea and highlighted by the extraordinary arrival of the virus on Spilsby Island.

**Figure 2:**
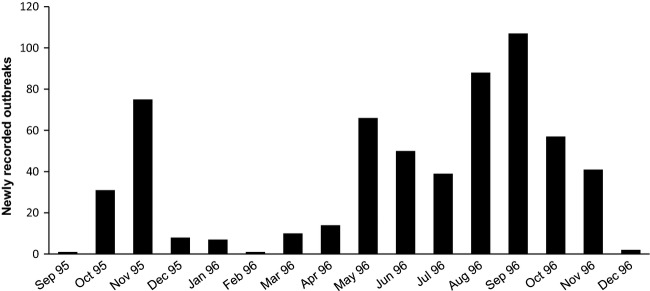
The counts of new rabbit haemorrhagic disease (RHD) outbreaks in South Australia in 1995/96. An outbreak was confirmed through the presence RHD viral RNA in recovered rabbit carcasses (see Kovaliski 1998). It shows that RHD was always active somewhere on a regional scale. The first record is the appearance of a dead rabbit outside the quarantine area on Wardang Island in September 1995.

The localities at which infected rabbits were found (Fig.1) by no means accounted for all rabbit populations in the region, but it is readily seen that virus from any given site where infected rabbits had been found could readily spread within a few weeks to almost any other part of the region considered. Collation of dates when RHD spread onto each site shows that RHD outbreaks were apparent in each month of the year (Fig.2). Nonetheless, most disease activity occurred from May to November with relatively few outbreaks recorded during the Australian summer and early autumn.

**Figure 3:**
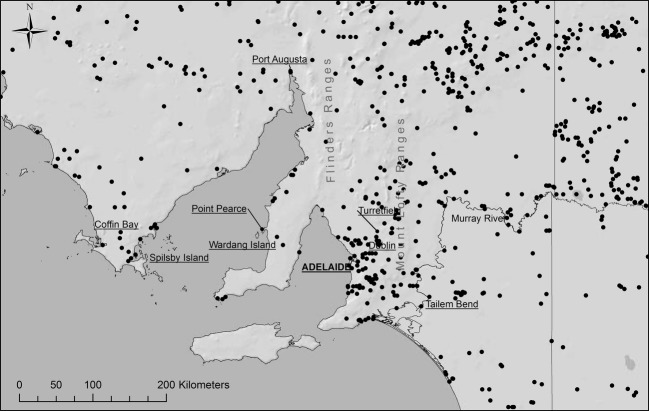
Map of presently known Southern Australian rabbit populations. Combined data from Rabbit Scan and databases on the first years of spread of rabbit haemorrhagic disease virus (RHDV). Each black dot represents a recorded rabbit population.

Virtually, all recorded populations of wild rabbits in the part of South Australia considered here are within 25 km of other known populations (Fig.3). This does not mean that there are no rabbits in between; instead, the data simply reflect the minimum density of rabbit subpopulations. No information about the structure of the individual rabbit populations is available.

Data so far indicates that RHD outbreaks can occur somewhere on a regional scale throughout the year even if at low, barely detectable levels over the summer months. This potentially allows RHDV to survive at a few localities on a wider regional scale and ensure its availability for spread back into local populations when conditions become favourable again. This is a feasible scenario, given the interconnectivity among rabbit populations, even over large distances, provided by flies or other flying vectors.

### Evidence for distinct virus clusters or genogroups in the Turretfield rabbit population

During our long-term study at Turretfield, rabbits that had died of RHDV were only found during a few weeks in spring and epizootics always started between August and October. These outbreaks were generally of short duration, and no rabbits confirmed to have died from RHDV were found at other times of the year.

**Figure 4:**
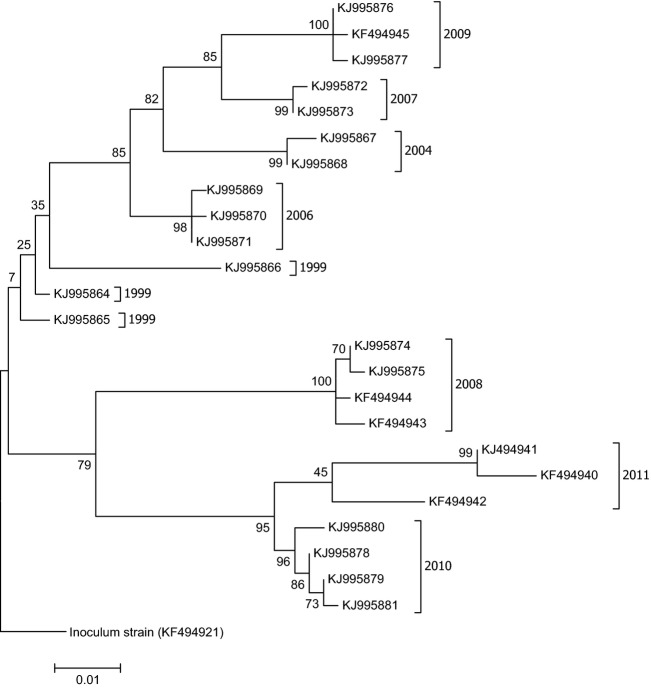
ML tree of rabbit haemorrhagic disease virus (RHDV) sequences (GenBank accession numbers) obtained from rabbits found dead at the Turretfield site (South Australia) between 1999 and 2011. Most variants cluster according to the year. The tree was rooted with the Australian RHDV inoculums strain that was manufactured from Czech CAPM-V351. It has been used since 1996 for biocontrol purpose and was in the same year deliberately released in the Turretfield population. Later, blood analyses showed, however, that the Turretfield population had already experienced a previous virus contact (most likely in 1995 when the virus escaped from an experimental site on Wardang Island, South Australia). Numbers at nodes represent bootstrap support (1000 iterations).

PCR confirmed the presence of RHDV RNA in 58 of the 63 samples available from dead rabbits collected at Turretfield. There was no evidence that individual rabbits had been infected by more than one virus variant. We identified 24 different RHDV sequences with 2–4 variants per year (Table2, for GenBank accession numbers: see Fig.4). The average number of nucleotide differences among RHDV variants obtained from the same year was 4.21 ± SE 4.45 (=0.9%). However, sequences from the years 1999 and 2011 showed a higher degree of variability than sequences in other years. Sequences isolated from rabbits that died in a given year clustered together (Fig.4). Exceptions were sequences from 1999, which were more diverse, and because of low bootstrap values, their phylogenetic position could not be resolved with confidence to be sure of their exact position in the dendrogram.

**Table 2:** Rabbit carcasses recovered from the Turretfield population.

Year	No of rabbits	RHDV positive	RHDV variants	*d*	SE	Proportion_*d*_
1999	15	15	3	10.67	2.55	0.023
2004	2	2	2	2.00	1.42	0.004
2006	6	6	3	1.33	0.89	0.003
2007	6	6	2	1.00	0.95	0.002
2008	8	8	4	2.67	1.12	0.006
2009	4	3	3	1.33	2.23	0.003
2010	19	15	4	2.67	1.15	0.006
2011	3	3	3	12.00	2.67	0.026
Sum	63	58	24			

Given are the number of rabbit carcasses tested for rabbit haemorrhagic disease virus (RHDV), the number tested positive for RHDV and the number of virus variants identified each year. The number of base differences averaged over all sequence pairs within each year (*d*) is shown with standard error estimate (SE). The proportion of *d* of the whole 465-bp fragment is given.

The phylogeny of virus sequences revealed two clusters. The first consisted of sequences obtained in the years 2004, 2006, 2007 and 2009. In 2005, no outbreak was detected. The other cluster included the sequences from 2008, 2010 and 2011. It is notable that sequences of successive years did not form sister groups. For example, sequences from 2009 were most closely related to those isolated in 2007 and not to those from 2008 – the latter even grouped in the second cluster, although with slightly lower statistical support. The only exceptions were the sequences from 2010 and 2011; they formed sister groups (Fig.4) and the position of one sequence from 2011 could not be clearly resolved as it was intermediate in sequence pattern.

## Discussion

Rabbits have a severe impact on the native Australian vegetation and fauna; they are associated with the spread of weeds and introduced plants. It has been estimated that a density threshold of approximately 0.5 rabbits ha^−1^ completely limits the regeneration of a large number of palatable native plant species, some of which play an essential role in ecosystem functioning (summarized by Cooke 2012). RHDV was initially highly successful in controlling rabbits, but they have regained abundance although not generally returning to premyxomatosis or pre-RHDV levels. Nonetheless, there is clear evidence that through co-evolutionary processes the efficiency of RHDV as a biocontrol agent is decreasing (Saunders et al. 2010). This means that rabbit populations remain widespread, even if sometimes not with high numbers of individuals and Cooke et al. (2010) reported that in south-eastern South Australia, rabbits were present on 70% of roadsides where uncleared patches of natural vegetation remained. As at Turretfield, not all populations are linked closely enough for rabbits to readily move between them but with vectors such as flies, which disperse widely and actively seek rabbit carcasses, most rabbit populations are likely to be highly connected in terms of virus spread. The localities of recorded South Australian rabbit subpopulations indicate that even the most widely spaced ones are no more than 25 km apart and it is clear that RHDV can be rapidly spread over the distances required to infect those subpopulations (15–60 km week^−1^).

The measured rates of virus spread were well beyond simple spread by rabbit to rabbit contact especially after allowing for a 2-day incubation period before infected rabbits begin to shed virus. Home ranges of individual wild rabbits are generally <400–600 m in diameter (Moseby et al. 2005; B. D. Cooke, unpublished data). This implies that flies are of key importance in the epidemiology of the disease, and there is a good case to argue that blowflies, such as *Calliphora spp*, are of particular importance (Barratt et al. 1998; Asgari et al. 1999) although specific studies of the distances calliphorid flies move on a daily or weekly basis are lacking.

Following Kovaliski et al. (2014), we independently show that there is little evidence to suggest that RHDV generally persists in the rabbit subpopulation at Turretfield from 1 year to the next. Instead, there is a stronger argument in favour of the virus being active somewhere on a wider regional level throughout the year but able to spread back through the Turretfield population when, as a result of breeding, there are sufficient susceptible subadult rabbits to carry an epizootic once more. It may be argued that this is tenuous because it is based on data from 1995 to 1996 as RHD first spread through naïve and highly susceptible rabbits. However, even in the contemporary, non-naïve Australian populations, young susceptible rabbits are available throughout much of the year on a regional scale, given the considerable seasonal variation in the length of the breeding period in Australian rabbit populations (Gilbert et al. 1987) and occasional unseasonal births after heavy summer rain. In more temperate, wetter regions of Australia, the continuous supply of susceptible rabbits could allow for outbreaks to extend later into the summer. For example, in 2010 and 2011, RHDV-positive carcass samples were collected in New South Wales and the Australian Capital Territory between February and April (Kovaliski et al. 2014).

On that basis, and knowing both that the rate of RHDV spread can be slow in summer (9 km month^−1^, Kovaliski 1998) and that viable virus can persist for some months in tissues within a cool burrow (McColl et al. 2002; Henning et al. 2005), we would expect the virus to survive the summer on a wider regional scale at least. This is important for understanding RHDV epidemiology in smaller, relatively isolated rabbit populations. Our phylogenetic analyses of virus variants isolated during the epidemics at Turretfield showed that the virus group seen in 1 year was usually replaced by a distinctly different group of virus variants in the following year's epizootic. The simplest explanation is that they were introduced afresh each year.

Even though RHDV appears to survive across the summer at a large geographic scale, possibly at relatively few localities, this does not rule out the possibility that it could occasionally persevere over summer at Turretfield or at a site nearby. In 2011, for example, the virus variants from Turretfield formed a sister group to the variants seen in 2010 but showed higher divergence within the group than other variant groups, with one variant being difficult to clearly assign to either the 2010 or 2011 virus cluster. This is expected given seasonal variation such as a longer breeding season and more prolonged availability of susceptible rabbits in wetter years. Indeed, 2010 and 2011 were the wettest years at Turretfield since 1992 (Australian Bureau of Meteorology), and rabbit breeding was extended as evidenced by the capture of rabbit kittens in January of both years. However, this does not seem to be an adequate explanation for the wide variability among RHDV variants collected in 1999 because there had been no outbreak of RHD in 1998. Higher variability had also been reported in the first years of spread and a possible explanation for this is that, up to that time, land managers were still making releases of Czech CAPM-V351 RHDV inocula to control their rabbits in Australia (Asgari et al. 1999). That could have led to wider initial genetic variation until interest in making further virus releases waned and a few well-adapted field variants of RHDV eventually became dominant.

Our data on nucleotide substitutions in RHD virus sequences across years in rabbits at Turretfield not only provide additional insights into the geographical scale needed to allow continuous virus circulation in the Australian rabbit meta-population but also parallel the long-term changes in epidemiology seen at Turretfield. In the first years of the study, RHD epidemics occurred irregularly, and in some years, the virus only appeared briefly on site (as recorded by some antibody seroconversions in live-trapped rabbits without rabbit carcasses being found) but did not cause an obvious disease outbreak. Since 2006, however, major outbreaks had occurred annually in sharp epizootics.

Our results enable us to say that, of the various scenarios proposed by Fouchet et al. (2009) to predict how virus virulence and rabbit resistance might co-evolve, observations in Australia align best with the scenario that those authors considered to be unlikely. Rather than viruses competing in small subpopulations of rabbits, our evidence supports the idea that RHDV is circulating through a very large, widespread rabbit meta-population and that transmission by vectors such as flies connects even spatially distant rabbit subpopulations. Under those circumstances, RHDV should be selected for high virulence, causing short strong outbreaks, but with low viral persistence probability within any given rabbit subpopulation (Fouchet et al. 2009).

Other recent work with nonpathogenic RCV-A1 in Australia offers a contrasting view, which accords better with the other scenarios put forward by Fouchet et al. (2009). This virus is nonpathogenic although eliciting a temporary antibody response (Strive et al. 2013) and seems to be transmitted by rabbit to rabbit contact. It has a limited distribution, generally in favourable climatic regions where rabbits produce at least some litters of young throughout most of the year, and it is known that two or more RCV-A1 variants from different clades or sister groups can be present in the same local rabbit population at a given time. They are likely to compete within those populations. Analyses of the most recent common ancestor (MRCA) of RCV-A1 genomes suggest that the virus arrived in Australia at about the time the first wild rabbits were imported from England in the late 1850s (Jahnke et al. 2010) confirming that the virus had persisted for the 2–3 month duration of the sea voyage. Extremely small host populations at that time may have further selected for locally persistent variants in RCV-A1. In contrast, RHDV spread over Australia after rabbits were well established, widespread and abundant.

Our envisaged model of RHDV circulation within a large meta-population, where virus variants are selected for their capacity to reach and rapidly infect rabbit subpopulations, has extremely important implications for future rabbit management within Australia. Apart from providing one of those rare occasions when epidemiological results from the field have proved useful in selecting an appropriate theoretical model, the combination of field data and theory helps explain why RHDV remains highly pathogenic and continues to cause high mortality in the field despite evidence that rabbits are developing some genetic resistance (Elsworth et al. 2012). The virus genome is changing rapidly and is clearly more efficient in the sense that the initial Czech CAPM-V351 virus only caused irregular outbreaks, whereas regular annual outbreaks of RHD have since become the pattern as the virus has become more diverse. Mutze et al. (2014) have further suggested that RHDV has evolved increased virulence recently as it now causes fatal disease in younger rabbits than was previously the case.

In planning future rabbit control in Australia, this growing theoretical framework is an important tool when it comes to decision making. The Invasive Animals Cooperative Research Centre is supporting projects in which RHDV variants imported from several countries are being assessed in quarantine to determine whether some may be more effective than current field variants in countering increasing rabbit numbers. While some of these candidate viruses cause high mortality in rabbits selected for resistance to Czech CAPM-V351, it is not a simple case of releasing such viruses without having some supporting theoretical framework to say that they are likely to retain their current virulence and effectively compete with existing field variants of RHDV. Our study provides a framework which should help to explore those future scenarios and judging risks and benefits if new RHDV variants were introduced into Australia.

For Europe, these ideas have a different significance because the main aim in Spain, Portugal and France was to reduce RHD impact on wild rabbit populations. One way of deciding whether RHDV is evolving to maintain high virulence, or whether it might be attenuating to less virulent forms, would be to sample RHDV within rabbit subpopulations and compare virus sequences from year to year. According to theoretical expectations, if there is evidence that several virus variants are competing within each rabbit subpopulation, this should result in attenuated, persistent viruses and lesser disease impact in the long run. Whether the interconnectivity through flies as vectors is less important in Europe and consequently results in a difference in selective pressure for virus virulence remains an important question to study.

Studies that investigate host–pathogen co-evolutionary processes of infectious diseases under natural conditions are important for many reasons other than the benefits they provide in biological control or because diseases cause problems for threatened wildlife, such as *O. cuniculus algirus* in Portugal and southern Spain. Pathogens have recently been recognized as an essential and inevitable part of all ecosystems with regulatory functions which can balance the biodiversity of an ecosystem (Lafferty 2014). In turn, the loss of biodiversity and habitat fragmentation is potentially associated with an increase in zoonotic and vector-borne disease outbreaks (Morand et al. 2014), and invasive pest species, such as rabbits and many other introduced vertebrates, do have a devastating impact on Australia's indigenous biodiversity. They are the major reason why in Australia nearly half the known mammalian extinctions worldwide in the past 200 years took place (summarized by Saunders et al. 2010). Host–pathogen co-evolutionary studies in wildlife species are therefore important in their own right from a scientific and epidemiological perspective, and understanding pathogen–host co-evolution within an epidemiological framework is an important aspect of evolutionary conservation.

## Data archiving statement

Dates, coordinates and test results of collected dead rabbits can be obtained from Biosecurity SA, GPO Box 1671, Adelaide, SA 5001, Australia. Information on the locations of recently recorded rabbit populations can be obtained from the Rabbit Scan project (http://www.feralscan.org.au/rabbitscan) and sequence data can be obtained from GenBank.

## References

[b1] Abrantes J, van der Loo W, Le Pendu J, Esteves P (2012). Rabbit haemorrhagic disease (RHD) and rabbit haemorrhagic disease virus (RHDV): a review. Veterinary Research.

[b2] Asgari S, Hardy JRE, Cooke BD (1999). Sequence analysis of rabbit haemorrhagic disease virus (RHDV) in Australia: alterations after its release. Archives of Virology.

[b3] Barratt BIP, Ferguson CM, Heath ACG, Evans AA, Logan RAS (1998).

[b4] Bowen Z, Read J (1998). Population and demographic patterns of rabbits (*Oryctolagus cuniculus*) at Roxby Downs in arid South Australia and the influence of rabbit haemorrhagic disease. Wildlife Research.

[b5] Cooke BD (2012). Rabbits: manageable environmental pests or participants in new Australian ecosystems?. Wildlife Research.

[b6] Cooke B, Jones R, Gong W (2010). An economic decision model of wild rabbit *Oryctolagus cuniculus* control to conserve Australian native vegetation. Wildlife Research.

[b7] Delibes-Mateos M, Delibes M, Ferreras P, Villafuerte R (2008). Key role of european rabbits in the conservation of the western Mediterranean basin hotspot. Conservation Biology.

[b8] Elsworth PG, Kovaliski J, Cooke BD (2012). Rabbit haemorrhagic disease: are Australian rabbits (*Oryctolagus cuniculus)* evolving resistance to infection with Czech CAPM 351 RHDV?. Epidemiology and Infection.

[b9] Fenner F, Fantini B (1999). Biological Control of Vertebrate Pests: The History of Myxomatosis, An Experiment in Evolution.

[b10] Forrester NL, Boag B, Moss SR, Turner SL, Trout RC, White PJ, Hudson PJ (2003). Long-term survival of New Zealand rabbit haemorrhagic disease virus RNA in wild rabbits, revealed by RT-PCR and phylogenetic analysis. Journal of General Virology.

[b11] Fouchet D, Le Pendu J, Guitton J-S, Guiserix M, Marchandeau S, Pontier D (2009). Evolution of microparasites in spatially and genetically structured host populations: the example of RHDV infecting rabbits. Journal of Theoretical Biology.

[b12] Gall A, Schirrmeier H (2006). Persistence of rabbit haemorrhagic disease virus genome in vaccinated rabbits after experimental infection. Journal of Veterinary Medicine, Series B.

[b13] Gall A, Hoffmann B, Teifke JP, Lange B, Schirrmeier H (2007). Persistence of viral RNA in rabbits which overcome an experimental RHDV infection detected by a highly sensitive multiplex real-time RT-PCR. Veterinary Microbiology.

[b14] Gilbert N, Myers K, Cooke BD, Dunsmore JD, Fullagar PJ, Gibb JA, King DR (1987). Comparative dynamics of Australasian rabbit-populations. Wildlife Research.

[b15] Gong W, Sinden J, Brasher M, Jones R (2009). The Economic Impact of Vertebrate Pests in Australia.

[b16] Henning J, Meers J, Davies PR, Morris RS (2005). Survival of rabbit haemorrhagic disease virus (RHDV) in the environment. Epidemiology and Infection.

[b17] Henzell RP, Cunningham RB, Neave HM (2002). Factors affecting the survival of Australien wild rabbits exposed to rabbit haemorrhagic disease. Wildlife Research.

[b18] Hicks A, Duffy S (2012). One misdated sequence of rabbit hemorrhagic disease virus prevents accurate estimation of its nucleotide substitution rate. BMC Evolutionary Biology.

[b19] Jahnke M, Holmes EC, Kerr PJ, Wright JD, Strive T (2010). Evolution and phylogeography of the nonpathogenic calicivirus RCV-A1 in wild rabbits in Australia. Journal of Virology.

[b20] Kerr PJ, Kitchen A, Holmes EC (2009). Origin and phylodynamics of rabbit hemorrhagic disease virus. Journal of Virology.

[b21] Kovaliski J (1998). Monitoring the spread of rabbit hemorrhagic disease virus as a new biological agent for control of wild European rabbits in Australia. Journal of Wildlife Diseases.

[b22] Kovaliski J, Sinclair R, Mutze G, Peacock D, Strive T, Abrantes J, Esteves PJ (2014). Molecular epidemiology of rabbit haemorrhagic disease virus in Australia: when one became many. Molecular Ecology.

[b23] Lafferty KD, Piña CI, Verdade LM, Lyra-Jorge MC (2014). Biodiversity loss and infectious diseases. Applied Ecology and Human Dimensions in Biological Conservation.

[b24] Liu J, Kerr PJ, Wright JD, Strive T (2012). Serological assays to discriminate rabbit haemorrhagic disease virus from Australian non-pathogenic rabbit calicivirus. Veterinary Microbiology.

[b25] McColl KA, Morrissy CJ, Collins BJ, Westbury HA (2002). Persistence of rabbit haemorrhagic disease virus in decomposing rabbit carcases. Australian Veterinary Journal.

[b26] Morand S, Jittapalapong S, Suputtamongkol Y, Abdullah MT, Huan TB (2014). Infectious diseases and their outbreaks in Asia-Pacific: biodiversity and its regulation loss matter. PLoS ONE.

[b27] Moseby KE, De Jong S, Munro N, Pieck A (2005). Home range, activity and habitat use of European rabbits (*Oryctolagus cuniculus*) in arid Australia: implications for control. Wildlife Research.

[b28] Moss SR, Turner SL, Trout RC, White PJ, Hudson PJ, Desai A, Armesto M (2002). Molecular epidemiology of *Rabbit haemorrhagic disease virus*. Journal of General Virology.

[b29] Mutze G, Cooke B, Alexander P (1998). The initial impact of rabbit hemorrhagic disease on European rabbit populations in South Australia. Journal of Wildlife Diseases.

[b30] Mutze GJ, Sinclair RG, Peacock DE, Capucci L, Kovaliski J (2014). Is increased juvenile infection the key to recovery of wild rabbit populations from the impact of rabbit haemorrhagic disease?. European Journal of Wildlife Research.

[b31] O'Hara P (2006). The illegal introduction of rabbit haemorrhagic disease into New Zealand. Revue scientifique et technique Office international des épizooties.

[b32] Ohlinger VF, Haas B, Meyers G, Weiland F, Thiel HJ (1990). Identification and characterization of the virus causing rabbit hemorrhagic disease. Journal of Virology.

[b33] Peacock D, Sinclair R (2009). Longevity record for a wild European rabbit, *Oryctolagus cuniculus*, from South Australia. Australian Mammalogy.

[b34] Saunders G, Cooke B, McColl K, Shine R, Peacock T (2010). Modern approaches for the biological control of vertebrate pests: an Australian perspective. Biological Control.

[b35] Shien JH, Shieh HK, Lee LH (2000). Experimental infections of rabbits with rabbit haemorrhagic disease virus monitored by polymerase chain reaction. Research in Veterinary Science.

[b36] Strive T, Elsworth P, Liu J, Wright J, Kovaliski J, Capucci L (2013). The non-pathogenic Australian rabbit calicivirus RCV-A1 provides temporal and partial cross protection to lethal rabbit haemorrhagic disease virus infection which is not dependent on antibody titres. Veterinary Research.

[b37] Tamura K, Stecher G, Peterson D, Filipski A, Kumar S (2013). MEGA6: molecular evolutionary genetics analysis version 6.0. Molecular Biology and Evolution.

[b38] Wang X, Xu F, Liu J, Gao B, Liu Y, Zhai Y, Ma J (2013). Atomic model of rabbit hemorrhagic disease virus by cryo-electron microscopy and crystallography. PLoS Pathogens.

[b39] Wardhaugh K, Rochester W (1996). Wardang Island. A retrospective analysis of weather conditions in relation to insect activity and displacement. Project CS 236. Australian & New Zealand Rabbit Calicivirus Disease Program – A Biological Control Initiative Against the European Wild Rabbit.

